# A Two-Stage Multi-Source Unilateral Alignment Method for Rail Insulation Fault Localization Under Target-Domain Missing-Class Conditions

**DOI:** 10.3390/s26175532

**Published:** 2026-08-31

**Authors:** Qiaoyue Li, Bo Chen, Guifu Du, Xiandong Li, Tongtong Zhou

**Affiliations:** 1 School of Optical and Electronic Information, Suzhou City University, Suzhou 215104, China; qyli@szcu.edu.cn (Q.L.); zhoutt@szcu.edu.cn (T.Z.); 2 School of Rail Transportation, Soochow University, Suzhou 215131, China; 20245246023@stu.suda.edu.cn; 3 School of Advanced Technology, Xi’an Jiaotong-Liverpool University, Suzhou 215123, China; xiandong.li23@student.xjtlu.edu.cn

**Keywords:** urban rail transit, insulation fault localization, target-domain training class incompleteness, multi-source domain transfer, unilateral alignment

## Abstract

To address the decline in insulation fault localization accuracy of urban rail DC traction return systems under varying operating conditions and missing target-domain classes, this study proposes a two-stage multi-source unilateral alignment network (MUAN). In practice, different loads and operating conditions lead to different rail-potential patterns, while the target-domain training set often lacks some fault classes. Conventional domain adaptation methods usually assume identical label spaces across domains, which may cause source-private classes to be incorrectly aligned and thus induce negative transfer. To mitigate this issue, a multi-source partial domain adaptation framework is developed. In the first stage, labeled source data from multiple conditions are used to learn discriminative features, and source anchor features are extracted for subsequent transfer. In the second stage, source-alignment and cross-domain alignment modules are introduced to guide the target features toward the shared class space while preserving the source class structure. Domain-adversarial learning is further employed to reduce cross-condition distribution gaps and improve generalization to unlabeled target data with missing classes. Experiments on both a dynamic rail-potential simulation platform and a return-system hardware platform show that the proposed method achieves superior fault localization performance, especially under target-domain missing-class settings.

## 1. Introduction

DC traction power supply systems are widely used in urban rail transit lines. They mainly consist of traction substations, overhead contact lines, trains, return conductors, and protection devices. Among them, the running rail not only supports train operation but also serves as the return path for traction current [1,2]. Because the running rail cannot be completely isolated from the ground, a portion of the traction current may escape into nearby conductive media, such as soil and underground metal structures, producing stray current. Together with the rail’s longitudinal resistance, this leakage gives rise to a voltage difference between the rail and the ground, referred to as rail potential [3,4]. When rail insulation degrades because of moisture, contamination, or aging of the ballast bed, fasteners, or insulators, stray current leakage increases and rail potential rises. Stray current may also enter nearby metallic structures, creating anodic regions where current leaves the structure and causing severe electrochemical corrosion, shortened service life, and higher maintenance costs. In addition, stray current can couple into signal circuits through the ground or other metallic loops, leading to track-circuit errors, signal distortion, and even serious train operation hazards such as false clear indications. Excessive rail potential may also pose electric shock risks, interfere with trackside equipment, and increase the risk of electrical faults or fire. Therefore, timely detection and localization of insulation faults is essential [5,6,7].

Due to the strong feature extraction capability of deep learning and its suitability for real-time online diagnosis, it has been widely applied in fault diagnosis [8,9]. In the field of urban rail fault diagnosis, Liu et al. [10] proposed a grounding-fault localization method for DC subway systems based on a multi-scale one-dimensional convolutional neural network (MS-1DCNN). Chen et al. [11] used 1DECA-SN, a ShuffleNet-based one-dimensional efficient channel attention network, to locate fault positions in running-rail grounding-fault scenarios while maintaining model lightweightness and practical applicability.

Most intelligent diagnostic models assume that the training and testing data are drawn from the same distribution. In real train operation, however, working conditions often change over time, which causes a noticeable shift between the source and target domains. To reduce the resulting performance drop, domain adaptation (DA) has been introduced. DA narrows the distribution gap across domains and helps the model maintain reliable diagnostic performance in new environments. In practice, it transfers knowledge from a labeled source domain to an unlabeled target domain through feature alignment [12,13]. Common DA methods include MMD, CORAL, DANN, and DSN [14,15,16,17].

To cope with performance loss caused by condition-dependent distribution shifts, many transfer diagnosis methods based on feature alignment have been developed. Li et al. [18] proposed a knowledge-mapping-based adversarial DA method that learns domain-invariant features through feature mapping and adversarial training, thereby improving generalization under varying operating conditions. Liu et al. [19] further proposed a Deep Adversarial Subdomain Adaptation Network, which extends global alignment to the subdomain level and improves fine-grained alignment between classes, reducing information loss caused by global matching alone. Jiang et al. [20] combined classifier-consistency constraints with self-supervised clustering in the target domain to enhance feature structure learning and improve performance under unlabeled target conditions. Yu et al. [21] integrated multi-scale feature extraction with multi-kernel maximum mean discrepancy (MK-MMD) to better align source and target distributions, improving stability and robustness under complex operating conditions.

Although these methods are effective in reducing cross-condition distribution shifts, most of them still assume that the source and target domains share the same label space. In practical industrial applications, especially in urban rail grounding-fault localization, data collection is limited by operating conditions, maintenance access, and fault occurrence rates. As a result, the target-domain training set often covers only part of the fault classes, and some states may not appear at all during sampling. In other words, the class space available in target-domain training is not identical to that of the source domain but forms only a subset of it. The source feature distribution therefore contains contributions from both shared and source-private classes, whereas the observable target distribution is mainly associated with the shared classes. Global alignment approaches, including MMD and CORAL, operate on statistics computed from the complete source and target distributions. As a consequence, source-private samples are also involved in the alignment, even though their corresponding classes are unavailable in the target-training data. In MMD, these samples contribute to the empirical mean embedding of the source distribution. In CORAL, they affect the covariance structure calculated over all source features. The resulting alignment may therefore move source-private features toward unrelated target feature regions without valid semantic correspondence. Such a mismatch can alter the discriminative structure between classes and eventually introduce negative transfer. For this reason, partial domain adaptation (PDA) has received increasing attention. Zhao et al. [22] proposed a Balanced and Weighted Alignment Network (BWAN), which jointly aligns marginal and conditional distributions through target-domain sample enhancement and source-domain weighting strategies, while suppressing the influence of source-private classes. Wang et al. [23] introduced an attention-based PDA method that employs a transferability evaluator and a cross-domain attention module to perform shared-class selection and weighted feature alignment, thereby improving transfer performance under missing-class conditions. Qin et al. [24] incorporated weighted MMD, weighted classification loss, and target-domain constraints into a Broad Learning System (BLS), reducing training complexity while maintaining transfer capability.

Although existing PDA methods can reduce negative transfer to some extent, most of them still project source and target features into a common latent space. Such bidirectional alignment strategies may also force source-private classes into the alignment process, causing feature confusion and weakening class discrimination when the target domain contains only partial categories. In addition, multiple source domains collected under different operating conditions are often available in practical applications, and these data contain complementary diagnostic knowledge that can further improve fault localization performance.

To address these limitations, a multi-source unilateral alignment strategy is proposed for rail insulation fault localization under PDA conditions. The source domains are used as alignment anchors, and only the target-domain features are guided toward the shared source-class space. This strategy preserves the discriminative structure of the source domain while reducing the influence of source-private classes. Based on this idea, a two-stage modular framework is developed, consisting of a domain-adversarial module (DANN), a source-domain alignment module (SDA), and a cross-domain alignment module (CDA). SDA is used to maintain stable source-domain feature relationships, whereas CDA refines target-domain features and progressively aligns them with the shared source-domain space. A further difference from conventional PDA settings is the treatment of missing classes at the testing stage. In many PDA tasks, the reduced target label space is maintained during both adaptation and testing. In the present study, classes are removed only from the target-training data, whereas the test set retains the full class space, including classes unseen during adaptation. This setting places a stronger requirement on the diagnostic model.

The main contributions of this study are summarized as follows:

(1) A two-stage modular unilateral alignment network (MUAN) is proposed for rail insulation fault localization, enabling accurate fault identification under multi-source and varying operating conditions.
(2) To address the missing-class problem in the target domain, a two-stage source-domain alignment module (SDA) and a cross-domain alignment module (CDA) are developed. These modules achieve unilateral target-to-source alignment while preserving the inter-class relationships of the source domain.
(3) Extensive partial cross-domain diagnosis tasks are conducted under different datasets and under different missing-class conditions to verify the effectiveness of the proposed method. Comparisons with several representative domain adaptation and PDA approaches further demonstrate the robustness and generalization capability of the proposed framework.

The rest of this paper is organized as follows. Section 2 introduces the background, including the urban rail DC traction power supply system, the dynamic rail-potential simulation platform, and the hardware experimental platform. Section 3 describes the proposed two-stage modular unilateral alignment framework (MUAN). Section 4 presents the experimental results and analysis. Section 5 concludes this paper.

## 2. Related Works

### 2.1. DC Traction Power Supply System

**Figure 1:**
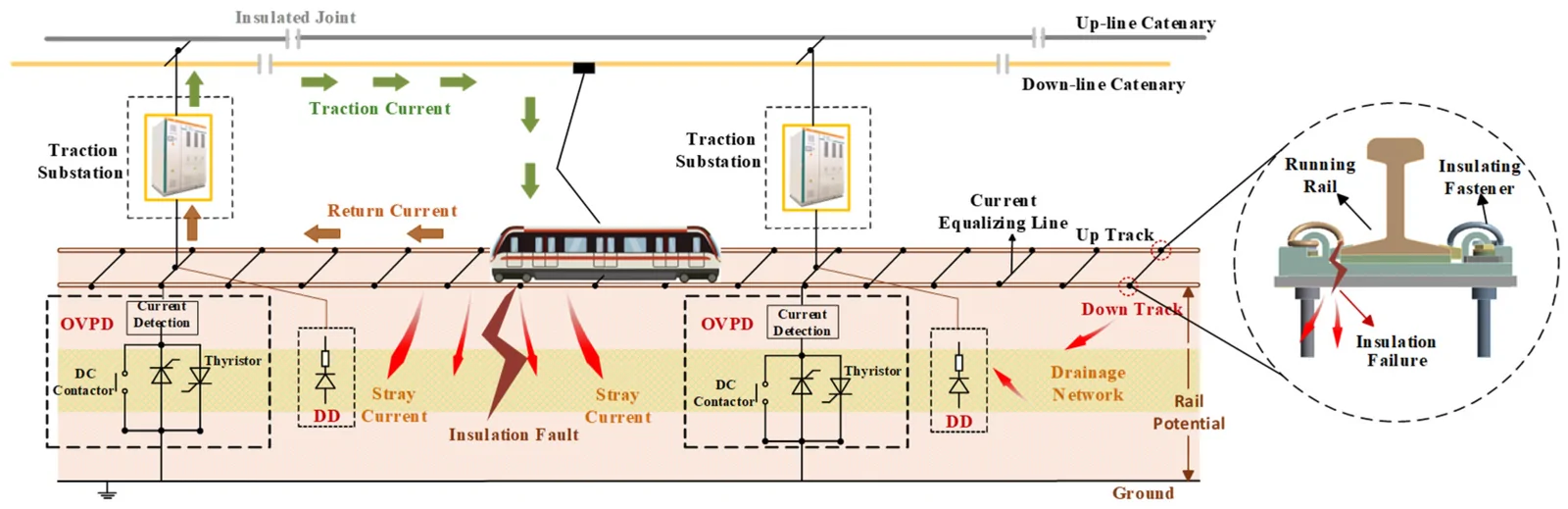
Schematic of the DC traction power network in urban rail systems.

Urban rail transit systems generally use DC traction power supply systems, which consist of traction substations, contact lines, trains, and return circuits. The traction substation includes rectifier units, regenerative energy absorption devices (READs), and auxiliary devices such as overvoltage protection devices (OVPDs) and drainage devices (DDs). The return circuit is mainly formed by running rails, drainage networks, and grounding grids. In practice, the running rail cannot be perfectly isolated from the ground, and a longitudinal resistance remains between the rail and the earth. This leads to rail potential and stray current. Environmental conditions can also modify the electrical behavior of the traction return system. Rail temperature directly affects longitudinal rail resistance because the resistivity of the rail material generally increases with temperature and decreases as the rail cools. These resistance variations alter current distribution along the return path and consequently influence rail-potential responses. Soil pH acts through a different mechanism. Rather than directly changing longitudinal rail resistance, it is associated with the electrical properties of ground-related leakage paths. Variations in pH may accompany changes in soil composition and ionic conduction, which can modify soil resistivity and the equivalent rail-to-ground leakage resistance. More conductive surrounding ground tends to provide a lower-resistance leakage path, increasing current leakage from the rail and affecting both rail potential and stray current. Lower ground conductivity produces the opposite tendency. The actual influence of pH depends on local soil composition, moisture content, and other environmental conditions. The drainage network collects leaked stray current, while the OVPD limits excessive rail-potential rise. The system layout is shown in Figure 1. When rail insulation degrades and grounding faults occur, the rail-to-ground resistance at the fault location drops sharply, which increases rail-potential fluctuations. When a train passes the fault section, the rail potential is often held near 0 V. Therefore, dynamic rail-potential signals during train operation are used as the input data for grounding-fault diagnosis in this study. In this study, however, domain differences are mainly introduced by changes in train load and the associated traction power. Environmental conditions are not explicitly defined as domain variables in either dataset. They are instead considered possible additional sources of distribution variation in practical railway operation.

### 2.2. Dynamic Rail-Potential Computation Platform

**Figure 2:**
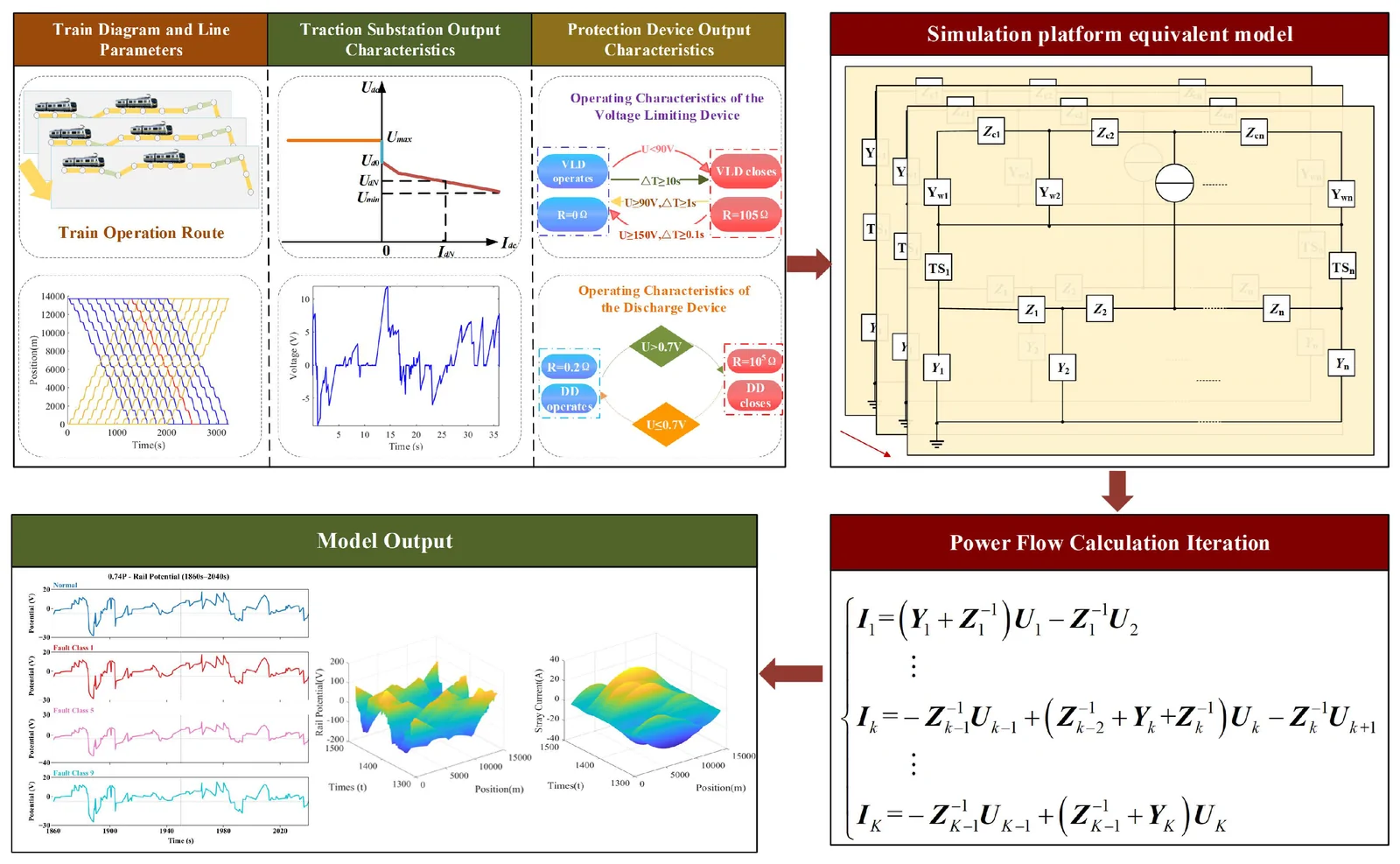
Schematic of the dynamic rail-potential simulation platform.

Based on the DC traction power supply system, a MATLAB-based dynamic simulation platform is developed to analyze the time-varying characteristics of rail potential under both normal and grounding-fault conditions. Separate equivalent models are established for healthy and fault states, and sectional divisions are introduced at traction substations, train positions, and fault locations to determine the model parameters through analytical calculation. The overall structure of the proposed simulation platform is illustrated in Figure 2. The input module mainly includes train operation schedules, line parameters, and the output characteristics of traction substations and protection devices. The train operation profile is obtained through traction calculation according to station spacing and real-time train conditions, from which the corresponding traction power and current at different time instants can be derived. During the equivalent modeling process, the electrical behavior of the system is represented by a double-π equivalent circuit. Different sectional division strategies are adopted for normal and fault conditions to ensure accurate parameter configuration and model representation. Based on the above approach, the dynamic distribution characteristics of rail potential under different operating and fault conditions can be effectively calculated and analyzed.

For the sections defined under normal insulation conditions, the voltage–current relationship between the running rail and the return network can be derived according to the distributed-parameter model of the return system, as expressed in Equation (1).

(1) $$\left\{\begin{array}{l} \frac{du_{r}(x)}{dx}=-R_{r}\cdot i_{r}(x)\\ \frac{du_{s}(x)}{dx}=-R_{s}\cdot i_{s}(x)\\ \frac{d{\dot{i}}_{r}(x)}{dx}=-G_{s}\cdot u_{r}(x)+G_{s}\cdot u_{s}(x)\\ \frac{d{\dot{i}}_{s}(x)}{dx}=G_{s}\cdot u_{r}(x)-(G_{p}+G_{s})\cdot u_{s}(x)\; \end{array}\right.$$

where *u_r_*, *u_s_*, *i_r,_* and *i_s_* denote the voltages and currents of the running rail and the drainage network, respectively. *R_r_* and *R_s_* represent the longitudinal resistance per unit length of the running rail and the drainage network. *G_s_* and *G_p_* denote the transition resistance per unit length between the running rail and the drainage network, and the equivalent ground resistance per unit length of the drainage network, respectively.

When a grounding fault occurs, a new section is introduced at the fault location, where *R_f_* is used to represent the equivalent rail-to-ground resistance. By adjusting *R_f_*, grounding faults with different severity levels can be modeled. Since this study focuses on locating insulation faults in different sections, sectional division and equivalent modeling are performed separately for each fault segment.

Based on the structural configuration of the traction power supply system and the electrical parameters of its components, the rail potential can be obtained through iterative power-flow calculation. To improve computational efficiency and reduce the burden of repeated iterations, the original system is equivalently represented by a unified chain model. During the iterative process, the rail potential is updated according to the equation GU=I. At the initial stage, the traction substation is assumed to operate under no-load conditions, and the corresponding node-voltage submatrix is initialized as $U_{k}^{\left(1\right)}={\left[U_{d0},\;U_{d0},\;0,\;0,\;0\right]}^{T}$. Based on the instantaneous traction power demand of the trains, the node current matrix $I_{k}^{\left(1\right)}$ and the nodal admittance matrix $G_{k}^{\left(1\right)}$ are constructed, after which the node voltage $U_{k}^{\left(2\right)}$ is updated using the corresponding calculation rule. Then, $I_{k}^{\left(2\right)}$ and $G_{k}^{\left(2\right)}$ are iteratively refreshed until the convergence criterion is satisfied. Specifically, the iteration stops when, after the *i*-th update, the voltage and power of all nodes meet the prescribed conditions $U^{\left(i\right)}-U^{\left(i-1\right)}<\varepsilon_{1}$ and $\Delta P^{\left(i\right)}<\varepsilon_{2}$. The unified chain representation avoids repeated reconstruction of the complete urban rail power-supply network during iteration. For a given simulation condition, the equivalent topology remains fixed within each time step, while train motion only requires the operating-state parameters to be updated. Hence, the extra computation introduced by dynamic operation mainly consists of state updating and convergence iterations rather than repeated network reconstruction. This keeps the computational demand within a manageable range. Through this iterative procedure, the rail-potential distribution under both healthy and grounding-fault conditions during train operation can be obtained. The resulting rail-potential distributions are then used as diagnostic samples to build datasets under different operating conditions for model training and performance evaluation.

### 2.3. Hardware Experimental Platform

**Figure 3:**
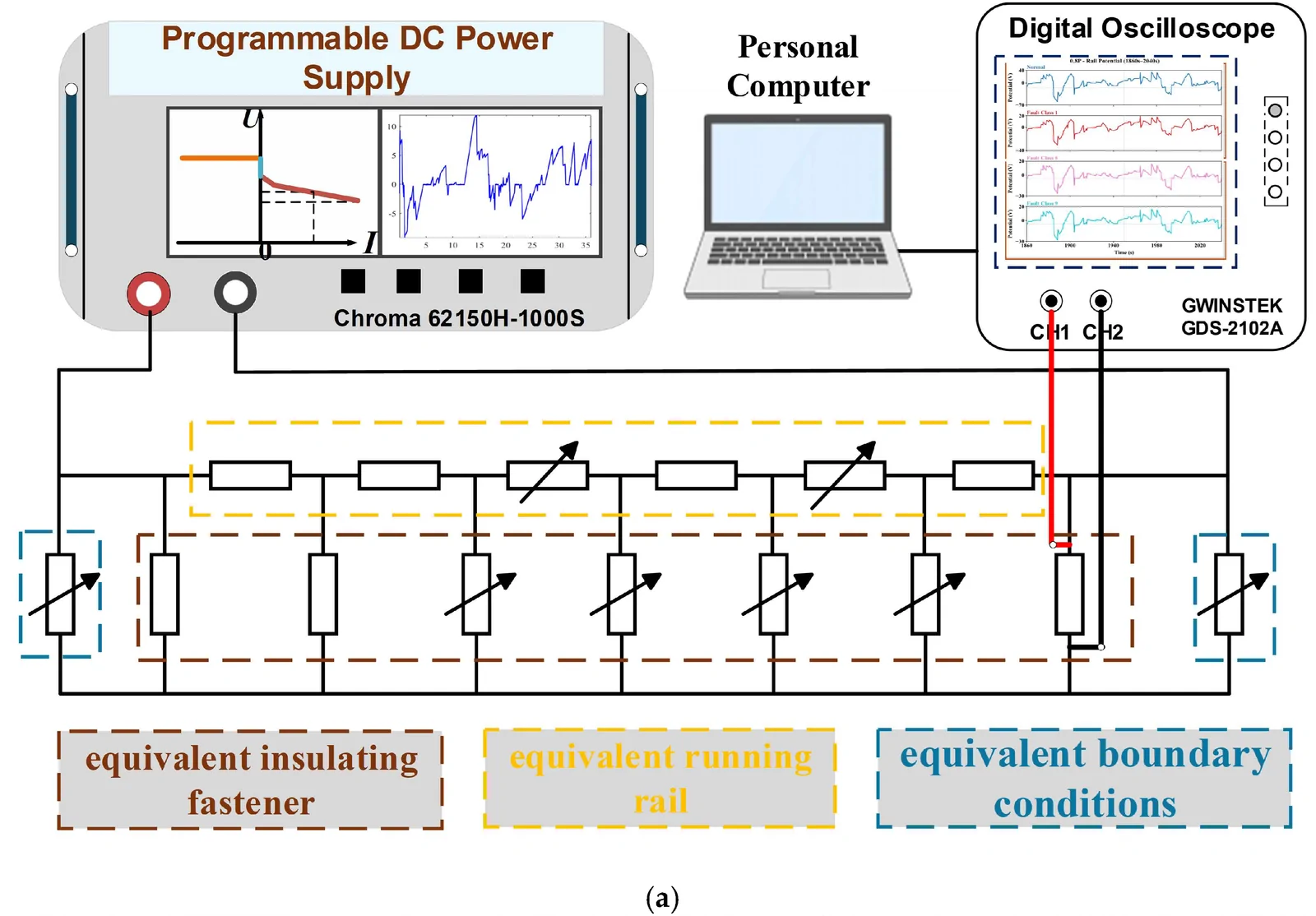

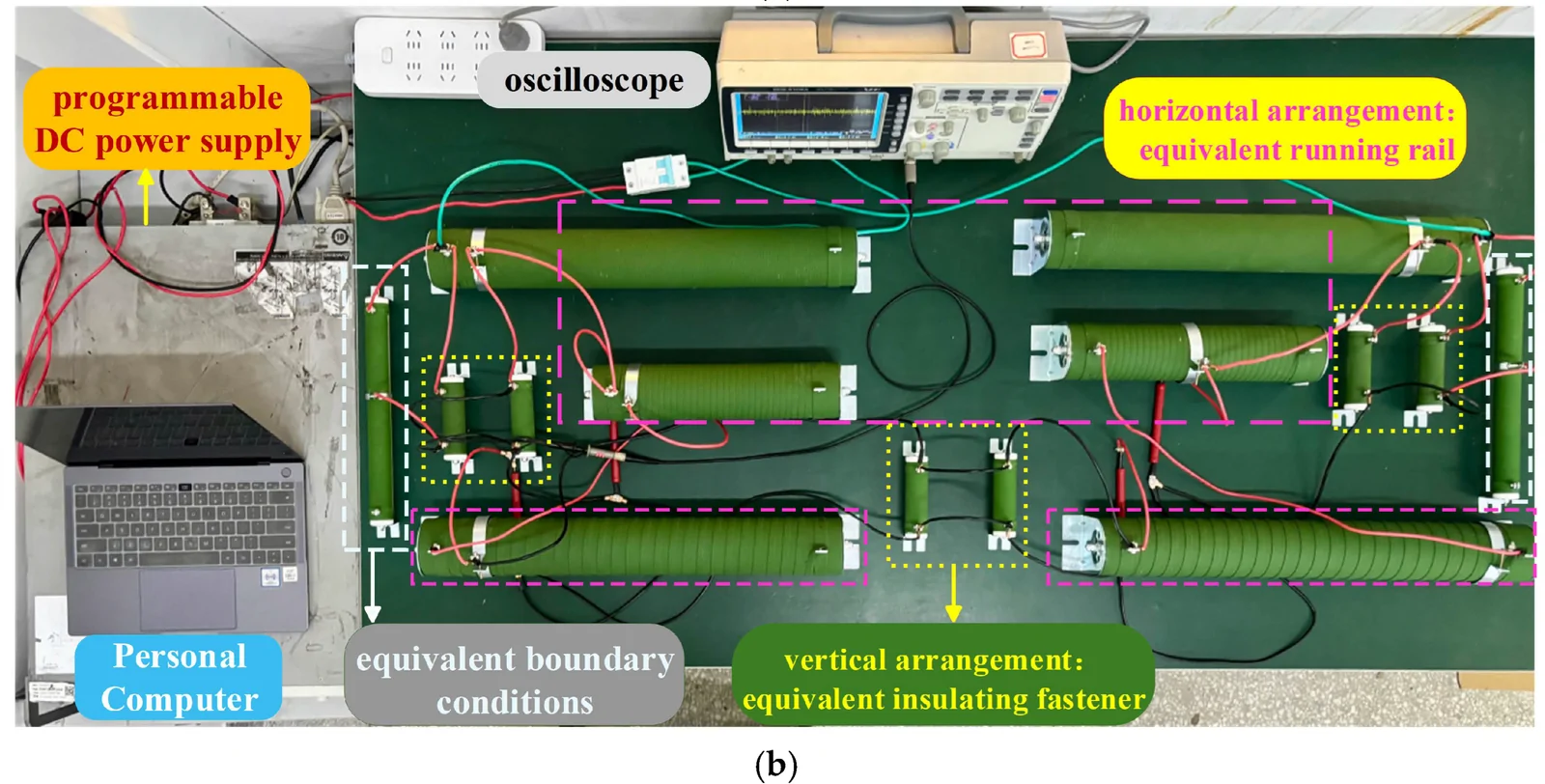
Schematic and actual setup of the return-system hardware experimental platform: (**a**) schematic diagram, (**b**) actual setup.

Considering that artificially introducing insulation faults in an actual urban rail return system is difficult to implement in practice, a hardware experimental platform was built in the laboratory in addition to the simulation-based dataset. This platform was used to reproduce the normal insulation state and grounding-fault state under different operating conditions, as shown in Figure 3. It mainly consists of a programmable DC power supply, adjustable resistor modules, a data recorder, and a personal computer. The programmable DC power supply provides the excitation current flowing through the simulated running rail. The adjustable resistors are used to model the rail characteristics, and their values are tuned to represent both healthy insulation and insulation failure conditions. The data recorder is used to measure and collect the rail-potential signals.

For parameter setting, six series resistors are used to emulate the actual running rail, with a longitudinal resistance of 0.02 Ω/m. In addition, seven resistors are connected in parallel on the equivalent rail to represent the rail-to-ground conductance, and each parallel branch is set to 1/15 Ω·m. When a grounding fault occurs in a certain section, the corresponding rail-to-ground conductance is increased to 1 × 10^3^ Ω·m. Moreover, the adjustable resistors at both ends are used to simulate the boundary conditions during train operation. By applying excitation sources at both ends of the equivalent circuit, the rail-potential distribution and stray-current behavior observed in real lines can be effectively reproduced, providing reliable data support for subsequent grounding-fault localization experiments. The RP signals measured on the hardware platform also contain high-frequency disturbances. Major sources include switching ripple and harmonics from the programmable DC power supply, measurement noise from the analog front end and A/D conversion, and disturbances introduced by connecting circuits. Electromagnetic interference from nearby laboratory equipment may also enter the measurement loop through signal cables. These components arise naturally from the experimental hardware and acquisition chain. No artificial noise was added to reproduce the exact spectral characteristics of an operating traction system. The hardware experiments therefore focus on robustness to practical measurement disturbances rather than exact reproduction of the field noise environment.

The hardware setup used here is a laboratory-scale equivalent system designed to reproduce representative electrical behavior of an urban rail return circuit under controlled conditions. It extends the numerical study by introducing practical experimental effects, including measurement noise and boundary variations. Nevertheless, it should not be interpreted as field validation on an operating metro line. Its role in this study is to examine MUAN under controlled hardware disturbances rather than to demonstrate direct deployment performance in a real railway system.

## 3. Proposed Method

The simulation and hardware platforms provide RP data under several operating conditions. However, changes in train load and traction power alter the statistical characteristics of these signals. A diagnostic model learned from one or more source conditions may therefore lose performance when transferred directly to an unseen operating condition. Another practical difficulty is that the target data available for adaptation may cover only part of the fault classes, even though the subsequent test data can contain the complete diagnostic class set. Under these conditions, global alignment may incorrectly associate target features with source regions belonging to classes that are absent during target-domain training, resulting in negative transfer.

**Figure 4:**
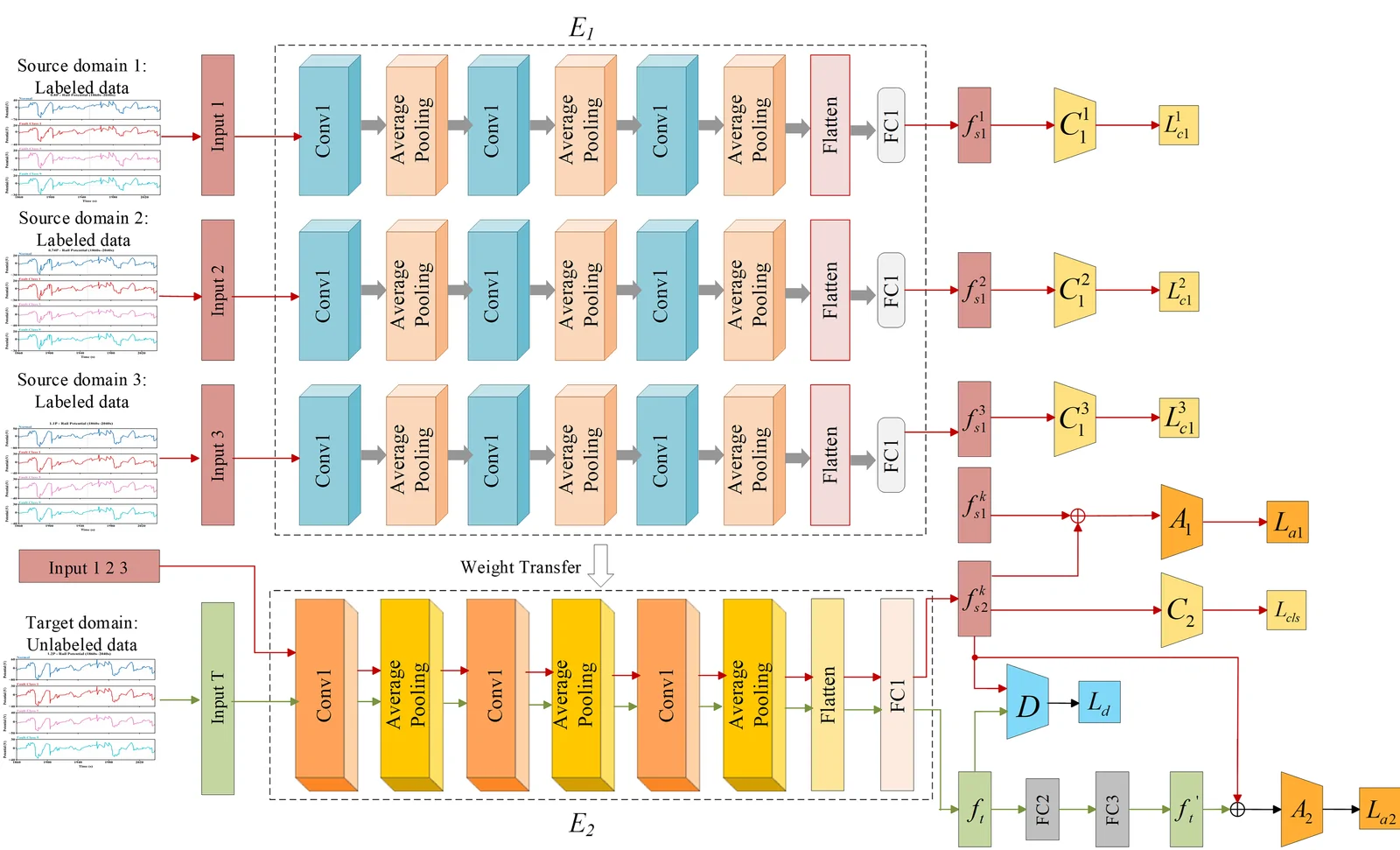
Architecture of the MUAN network.

To overcome these limitations, this study develops a new multi-source partial domain adaptation method, referred to as a two-stage modular unilateral alignment network (MUAN), as illustrated in Figure 4. The framework includes a multi-source pretraining stage and a multi-source weighted unilateral alignment stage. It is composed of a domain-adversarial module (DANN), a two-stage source-domain alignment module (SDA), and a cross-domain alignment module (CDA). Specifically, the DANN module includes two feature extractors *E*_1_ and *E*_2_, two classifiers *C*_1_ and *C*_2_, and a domain discriminator *D*. With this modular structure, the source-domain inter-class organization is kept stable while target representations are guided toward the multi-source feature space. This improves both robustness and localization accuracy when the target-training set is incomplete in class coverage. The detailed model parameters are listed in Table 1.

### 3.1. Problem Formulation

Let the source domain consist of *K* datasets collected under different operating conditions, denoted as $D_{s}=\{D_{s}^{1},D_{s}^{2},\ldots ,D_{s}^{K}\}$. The *k*-th source-domain dataset is defined as $D_{s}^{k}={\left\{(x_{s,i}^{k},y_{s,i}^{k})\right\}}_{i=1}^{n_{s}^{k}}$, where $x_{s,i}^{k}$ is the *i*-th sample in the *k*-th source domain, $y_{s,i}^{k}$ is the corresponding fault label, and $n_{s}^{k}$ is the number of samples in that domain. Since the source domains are collected under different working conditions, their data distributions differ, i.e., $P_{s}^{1}(x)\neq P_{s}^{2}(x)\neq \cdots \neq P_{s}^{K}(x)$. The target-domain dataset is denoted as $D_{t}={\left\{x_{t,j}\right\}}_{j=1}^{n_{t}}$, which contains only unlabeled samples and follows the target distribution *P_t_*(*x*). More specifically, the relationship between the source-domain label space, the target-domain training label space, and the target-domain test label space can be expressed as $C_{t}^{\mathrm{train}}=C_{s}\setminus M$, whereas $C_{t}^{\mathrm{test}}=C_{s}$.

Under this setting, conventional domain adaptation methods may transfer source-private classes to the target domain and thus cause negative transfer. MUAN therefore adopts a two-stage strategy consisting of multi-source pretraining followed by unilateral alignment. The first part preserves a stable source feature structure, while the second moves target representations toward this structure and limits interference from source-private classes. During adaptation, no information is provided about which target classes are absent. The missing-class identities are used only when constructing the experimental settings and evaluating the results. Thus, explicit identification of source-private classes does not rely on target labels.

### 3.2. Stage 1: Multi-Source Pretraining and Anchor Feature Construction

To obtain stable class-discriminative features from each source domain and provide a reliable reference for the subsequent unilateral alignment stage, supervised pretraining is performed separately on each source domain in the multi-source setting. This design preserves both intra-class compactness and inter-class separability within each source domain. As a result, Stage 2 no longer relies on a single source domain. Instead, it uses an anchor set formed by multiple pretrained source domains as the transfer basis, which improves the model’s adaptability to different operating conditions. For the *k*-th source domain, an auxiliary network is built with a feature extractor $E_{1}^{k}(\cdot )$ and a classifier $C_{1}^{k}(\cdot )$, whose parameters are denoted by $\theta_{e1}^{k}$ and $\theta_{c1}^{k}$, respectively. During pretraining, the network is optimized with the standard cross-entropy loss, as shown in Equation (2).

(2) $$L_{pre}^{k}=-\frac{1}{n_{s}^{k}}\sum_{i=1}^{n_{s}^{k}}\sum_{c=1}^{\left|C_{s}\right|}I(y_{s,i}^{k}=c)\log\frac{\exp(z_{i,c}^{k})}{\sum_{m=1}^{\left|C_{s}\right|}\exp(z_{i,m}^{k})}$$

Here, I(·) is the indicator function, and $z_{i,c}^{k}$ is the output score of sample $x_{s,i}^{k}$ for class *c* accordingly. The optimization objective of Stage 1 can then be written as Equation (3).

(3) $$({\hat{\theta }}_{e1}^{k},{\hat{\theta }}_{c1}^{k})=\arg\min_{\theta_{e1}^{k},\theta_{c1}^{k}}L_{pre}^{k}$$

After pretraining, the auxiliary network parameters are frozen, and the anchor features of each source domain are extracted, as shown in Equation (4).

(4) $$f_{s,i}^{k}=E_{1}^{k}(x_{s,i}^{k})$$

Since only labeled source data are used in this stage, the extracted features retain the original discriminative structure of the source domains and can serve as a reference for target-domain correction in the next stage. To further strengthen the representation of multi-source knowledge, a class prototype can also be constructed for each source domain, as given in Equation (5).

(5) $$\mu_{c}^{k}=\frac{1}{\left|D_{s,c}^{k}\right|}\sum_{(x,y)\in D_{s,c}^{k}}E_{1}^{k}(x)$$

where $D_{s,c}^{k}$ denotes the sample set of class *c* in the *k*-th source domain. $\mu_{c}^{k}$ denotes the class prototype, which gives a compact description of the discriminative class structure learned in Stage 1. In the current implementation, these prototypes are neither trainable variables nor components of an explicit prototype-level loss in Stage 2. They are used only to summarize class-level source characteristics. The actual structural reference for source consistency is provided by the pretrained source anchor features extracted in Stage 1. Accordingly, the prototypes serve mainly as descriptive class-level representations, whereas Stage 2 directly uses the pretrained anchor features together with the corresponding feature-level alignment loss.

### 3.3. Stage 2: Multi-Source Weighted Unilateral Alignment

In Stage 2, a main network is developed under the adversarial framework of GANs to realize directional transfer of target-domain features toward the shared source-class space. The network mainly consists of a feature extractor *E*_2_, a classifier *C*_2_, a domain discriminator *D*, and a target-domain feature correction module *G*.

#### 3.3.1. Source-Domain Alignment Module

Each source domain contributes to the multi-source adaptation process through a weight *w_i_*. In the present implementation, equal weights are assigned before training instead of being learned jointly with the network or estimated from source–target similarity. For a task involving *K* source domains, the weight of source domain *i* is defined as

(6) $$w_{i}=\frac{1}{K}=\frac{1}{3},\;\;i=1,2,3$$

and satisfies

(7) $$\sum_{i=1}^{K}w_{i}=1$$

Since three source domains are used in each transfer task, *w_i_* = 1/3 for all sources.

The weights remain unchanged throughout training. They are not updated by backpropagation and are independent of training iteration, parameter evolution, or source–target similarity.

For samples from multiple source domains, the classification loss with source weighting is defined in Equation (8).

(8) $$L_{cls}=-\sum_{k=1}^{K}\omega_{k}\frac{1}{n_{s}^{k}}\sum_{i=1}^{n_{s}^{k}}\sum_{c=1}^{\left|C_{s}\right|}I(y_{s,i}^{k}=c)\log\frac{\exp({\tilde{z}}_{i,c}^{k})}{\sum_{m=1}^{\left|C_{s}\right|}\exp({\tilde{z}}_{i,m}^{k})}$$

where ${\tilde{z}}_{i,c}^{k}$ are the output scores of the main network for source samples.

When domain-adversarial training disturbs the source feature distribution, the SDA module constrains the Stage-2 source features to remain close to the stable class-discriminative features learned in Stage 1. This alignment is carried out in two steps. First, a CNN-based feature extractor $E_{1}^{k}(\cdot )$ is trained in Stage 1, and source-only features are extracted as $f_{s,i}^{k}=E_{1}^{k}(x_{s,i}^{k})$. Then, in Stage 2, the weights of *E*_1_ are transferred to initialize *E*_2_. The source features $f_{s,i}^{k}=E_{2}^{k}(x_{s,i}^{k})$ are extracted together with target samples. To avoid damaging the source feature structure learned in Stage 1, a source consistency loss is introduced, as shown in Equation (9).

(9) $$L_{a1}=\sum_{k=1}^{K}\omega_{k}\frac{1}{n_{s}^{k}}\sum_{i=1}^{n_{s}^{k}}{\left\|E_{2}(x_{s,i}^{k})-E_{1}^{k}(x_{s,i}^{k})\right\|}_{1}$$

By minimizing Equation (9), the feature distribution learned in Stage 2 is forced to remain close to the pretrained source distribution, which helps stabilize the source-class structure. The L1 distance measures the dimension-wise deviation between the Stage 2 source features and their Stage 1 anchors. This form directly serves the purpose of retaining the pretrained source representation during adaptation. Unlike L2, which amplifies large deviations through a quadratic penalty, L1 increases linearly with the feature difference. It is therefore less dominated by a few dimensions that may change substantially during adversarial optimization. Cosine similarity, by comparison, mainly preserves feature direction and is less sensitive to magnitude variation. Since the source consistency term is intended to constrain both the dimension-wise representation and its magnitude relative to the pretrained anchors, L1 is adopted in this work.

#### 3.3.2. Target-Domain Alignment Module

Similar to SDA, a target-domain unilateral alignment module is designed to handle the cross-domain shift of the target domain relative to the source domains. For target samples, the feature extractor first produces the raw feature representation $f_{t,j}=E_{2}(x_{t,j})$. The target correction module *G* is then applied to refine these features, as shown in ${\tilde{f}}_{t,j}=G(f_{t,j})$. The module *G* is composed of two fully connected layers and is used to compensate for the distribution shift caused by domain mismatch. To align the target-domain features with a stable multi-source feature structure, a weighted multi-source cross-domain alignment term is defined. First, the multi-source source-feature center is constructed as in Equation (10).

(10) $${\bar{f}}_{s}=\sum_{k=1}^{K}\omega_{k}\left(\frac{1}{n_{s}^{k}}\sum_{i=1}^{n_{s}^{k}}\phi (E_{2}(x_{s,i}^{k}))\right)$$

Then, the corrected target-feature center is defined in Equation (11).

(11) $${\bar{f}}_{t}=\frac{1}{n_{t}}\sum_{j=1}^{n_{t}}\phi ({\tilde{f}}_{t,j})$$

The corresponding multi-source cross-domain alignment loss is given in Equation (12).

(12) $$L_{a2}={\left\|{\bar{f}}_{s}-{\bar{f}}_{t}\right\|}_{H_{\kappa }}^{2}$$

where *ϕ*(⋅) denotes the feature mapping function and *H_k_* is the reproducing kernel Hilbert space associated with kernel κ. A Gaussian radial basis function (RBF) is used for kernel κ. Its nonlinear mapping allows the alignment term to capture complex discrepancies between deep source and target representations, which is suitable for the objective of CDA. The kernel is written as

(13) $$\kappa (x_{i},x_{j})=\exp\left(-\frac{{\left\|x_{i}-x_{j}\right\|}_{2}^{2}}{2\sigma^{2}}\right)$$

The bandwidth σ is determined adaptively from the median pairwise Euclidean distance of the feature samples in each mini-batch.

By minimizing Equation (12), the discrepancy between the source feature distribution and the corrected target distribution is reduced. In this way, the target domain is pulled toward the multi-source anchor space without significantly disturbing the original source discriminability.

### 3.4. Optimization Objective and Algorithm Implementation

After enforcing source consistency and target unilateral correction, a domain discriminator is introduced to further reduce the residual source–target distribution gap through adversarial learning. The corresponding domain-adversarial loss is given in Equation (14).

(14) $$L_{d}=\sum_{k=1}^{K}\omega_{k}\left(\frac{1}{n_{s}^{k}}\sum_{i=1}^{n_{s}^{k}}D(E_{2}(x_{s,i}^{k}))-\frac{1}{n_{t}}\sum_{j=1}^{n_{t}}D(E_{2}(x_{t,j}))\right)$$

The final training objective of the whole model is written in Equation (15).

(15) $$L_{total}=L_{cls}+\alpha (L_{a1}+L_{a2})+\beta L_{d}$$

where α and β are balancing coefficients that control the contribution of different loss terms. Therefore, the parameter optimization targets can be expressed as Equations (16) and (17).

(16) $$({\hat{\theta }}_{e2},{\hat{\theta }}_{c2},{\hat{\theta }}_{g})=\arg\min_{\theta_{e2},\theta_{c2},\theta_{g}}L_{total}$$

(17) $${\hat{\theta }}_{d}=\arg\max_{\theta_{d}}L_{total}$$

During training, the feature extractor *E*_2_, classifier *C*_2_ and correction module *G* are updated by minimizing the total loss, so that the source–target distribution gap is gradually reduced. Meanwhile, the domain discriminator *D* is optimized in the adversarial direction to make domain discrimination harder and thus encourage domain-invariant feature learning. Through this joint optimization, the source discriminative structure is preserved, while target features are effectively transferred toward the source-class space. As a result, cross-domain fault diagnosis performance is improved under multi-source, varying conditions with missing target-domain classes.

Algorithm flow

Stage 1: Multi-source pretraining

First, the labeled samples from each source domain are fed into the corresponding auxiliary network $E_{1}^{k}$ and $C_{1}^{k}$. The class-discriminative features of each source domain are learned by minimizing the cross-entropy loss in Equation (2). Once pretraining is completed, the auxiliary network is frozen, and the extracted source anchors are retained for Stage 2. Class prototypes are obtained by averaging pretrained features within each class and are used only as auxiliary descriptions of the source class structure rather than as optimization terms.

Stage 2: Multi-source unilateral alignment

In Stage 2, the source samples and unlabeled target samples are jointly fed into the main network *E*_2_, *C*_2_, *G*, and *D*. The source samples are supervised by the classification loss *L_cls_*, while the source consistency loss *L_a_*_1_ keeps the source feature structure stable. The target samples are first corrected by module *G* and then aligned toward the multi-source feature space using the cross-domain loss *L_a_*_2_. The domain discriminator further reduces the distribution mismatch through the adversarial loss *L_d_*. Finally, the parameters of the feature extractor, classifier, and correction module are updated by minimizing *L_total_*, while the domain discriminator is updated in the opposite direction. This design makes full use of multi-source labeled information and avoids incorrect bidirectional alignment under missing target classes.

## 4. Case Study

### 4.1. Dataset and Task Description

The dataset was established using operational data from a metro line and a dynamic rail-potential simulation platform. Rail-potential signals over one complete power cycle were selected as samples due to their periodic characteristics. Monitoring points were placed at 6199 m and 6400 m. Samples were collected from 1860 s to 2040 s with a step size of 0.5 s, resulting in 720 points per sample. As shown in Table 2, the line was partitioned into 20 m fault localization sections, forming ten classes: one healthy class (class 0) and nine fault classes (classes 1–9). Under normal insulation conditions, *R_r_* is set to 0.02 Ω/km, and *G_s_* is set to 15 Ω⋅km. When an insulation fault occurs, *G_s_* at the fault location is reduced to 1 Ω. Each fault type contained 100 samples, and the dataset size for each operating condition was therefore 1000 × 720.

Based on the actual train operating conditions, four operating scenarios were further considered: no load, rated load, full load, and overload. The traction force *F* under different load conditions was calculated using Equation (18).

(18) $$F=9.81G(102(1+\lambda )\times a+f_{x}+f_{p}+f_{y}+f_{c}$$

**Figure 5:**
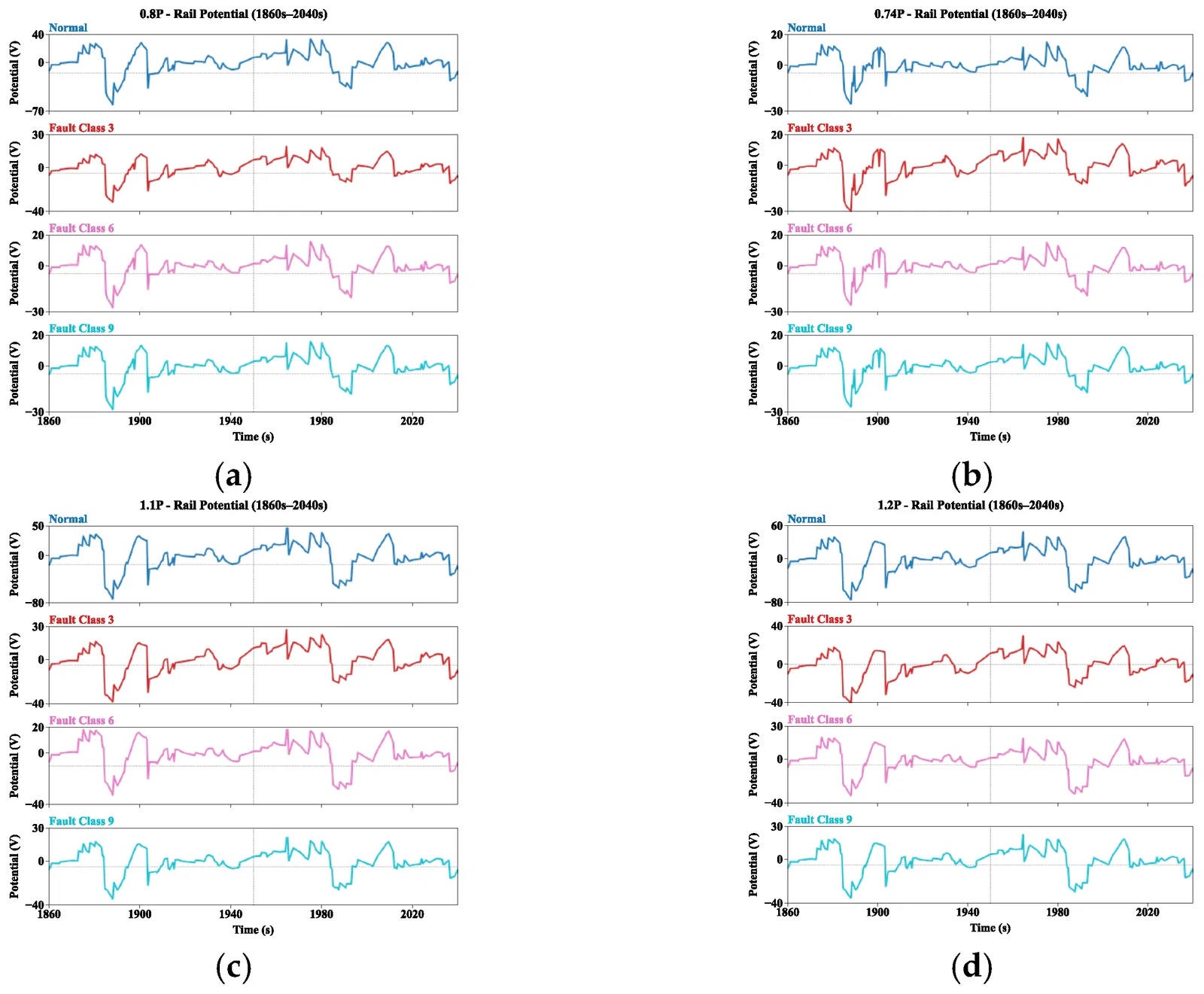
Raw rail-potential (RP) signals of selected labels (0, 3, 6, 9) under four operating conditions: (**a**) 0.8P, (**b**) 0.74P, (**c**) 1.1P, (**d**) 1.2P.

Here, *G* is the train mass, set to 300 t; λ is the inertia coefficient of rotating components, set to 0.1; and *a* is the train acceleration, typically ranging from 0.9 to 1.0 m/s^2^. The terms *f_x_*, *f_p_*, *f_y,_* and *f_c_* denote the basic resistance, grade resistance, curve resistance, and tunnel resistance, respectively. Based on the traction calculation, the train traction power is obtained as *P* = 3507.39 kW. Accordingly, the traction powers for the four operating conditions were set to 0.74P, 0.8P, 1.1P, and 1.2P. These conditions were then used to construct the source and target domain datasets. The corresponding RP signals of different fault sections under the four operating conditions are shown in Figure 5.

In addition, experiments were conducted using the return-system hardware platform described in Section 2.3. The boundary conditions obtained from the simulation platform were used to reproduce variations in rail potential (RP) and stray current (SC) under controlled conditions. Insulation faults at different sections were emulated by adjusting the resistor modules. The equivalent line covered 5800–6867 m, with 5900–6080 m defined as the fault region. RP monitoring points were placed at 5900 m and 6100 m. The RP signals in the hardware experiments were acquired using a GWINSTEK GDS-2102A at a sampling frequency of 1000 Hz. The raw signals were first processed using a 50 Hz low-pass filter. Two RP monitoring points were used, and after downsampling, 360 data points were retained for each monitoring point. The signals from the two monitoring points were then combined to form one diagnostic sample, resulting in 720 data points per sample. Under each identical combination of power-supply condition, resistance configuration, and fault location, the acquisition procedure was independently repeated three times. For each 20 m fault localization section, the equivalent insulation-fault position was varied at intervals of approximately 0.6 m according to the spacing between adjacent rail fasteners. Independent RP measurements were repeatedly acquired until 100 valid samples were obtained for each fault class. These samples were not generated by segmenting, sliding-window sampling, or duplicating a smaller number of long experimental records. As in the simulation platform, four operating conditions (0.8P, 0.74P, 1.1P, and 1.2P) were considered. It should be emphasized that this dataset is not field data collected from an operating metro line with naturally occurring insulation faults. The hardware results are mainly used to evaluate the robustness and transfer capability of the proposed method under controlled experimental conditions.

For these four operating conditions, RP data were collected from both the simulation platform and the hardware platform. The four conditions were then alternately used as target domains, while the remaining three were treated as source domains, thereby forming multi-source cross-domain diagnosis tasks. The distribution shifts considered in this study mainly arise from cross-condition variations in train load and traction power rather than from the inherent feature differences between the healthy state and different fault classes.

Within each operating condition, samples from every class were first split into a 70% training portion and a 30% independent test portion. This gave 70 training samples and 30 test samples per class. For each source domain, the 70 training samples were further separated into 63 samples for optimization and 7 samples for validation, giving 630 training and 70 validation samples per source domain. The target-training portion was treated as unlabeled adaptation data. A predefined set *M* was used to generate the missing-class scenarios. If *c* ∈ *M*, all 70 target-training samples belonging to class *c* were excluded from adaptation. The target adaptation set therefore contained 70(10 − ∣*M*∣) samples. The model received no information about which classes had been removed. Importantly, the removal applied only to the adaptation set. The final target test set retained all ten classes, with 30 samples per class and 300 samples in total.

No labeled target samples were involved in validation, model selection, or hyperparameter tuning, thereby preventing target-label leakage and preserving the unsupervised adaptation setting. Only the source validation set was used to monitor training and select models. The source training set, source validation set, target adaptation set, and final test set contained no overlapping samples. In particular, test samples were never used during training, adaptation, validation, model selection, or hyperparameter tuning.

Three representative missing-class patterns were used to examine adaptation under different forms of target-training incompleteness. Classes 3–6 formed a moderate continuous gap involving several neighboring fault sections. Classes 1, 3, 6, and 8 formed a discrete pattern distributed across separated locations. Classes 2–7 represented a severe continuous case in which six of the nine fault classes were unavailable during adaptation. These settings covered different missing-class structures and severities rather than exhaustively enumerating every possible combination. Each experiment was repeated five times under the same data split but with independent random initialization and training randomness. Results are reported as the mean ± standard deviation over these runs.

The signals were then normalized to the range [−1, 1] using the Min–Max method:

(19) $$x\prime =2\times \frac{x-x_{\min}}{x_{\max}-x_{\min}}-1$$

where *x* is the raw voltage signal collected from the two monitoring points under a given insulation condition, *x*′ is the normalized RP signal, and *x_min_* and *x_max_* are the minimum and maximum values of the sample, respectively. This normalization helps reduce amplitude differences across operating conditions and improves training stability and convergence speed. For the simulation-based dataset, since no obvious high-frequency noise was present, only Min–Max normalization was applied before model input.

### 4.2. Comparative Methods and Parameter Settings

To assess the proposed MUAN under target-domain label-space incompleteness, four widely used transfer diagnosis approaches, namely DANN, MDFAN, BWAN, and PADA, were selected as benchmarks. These methods represent different adaptation paradigms, including adversarial adaptation, multi-source adaptation, weighted transfer, and partial domain adaptation, providing a comprehensive basis for performance evaluation under multi-source and missing-class scenarios:

(1) DANN: DANN is a representative adversarial domain adaptation framework. It employs a domain discriminator and gradient reversal learning to minimize domain discrepancy and enhance feature transferability. The model consists of a feature extractor, a classifier, and a domain discriminator. Source-domain labels are used for classification learning, while adversarial optimization encourages the extraction of domain-invariant representations [25,26].
(2) MDFAN: MDFAN is a multi-source domain adaptation approach developed for cross-condition fault diagnosis. The framework combines feature extraction with multi-domain adaptation to reduce distribution discrepancies among heterogeneous operating conditions. Meanwhile, a classifier coordination strategy is employed to improve prediction consistency and strengthen diagnostic performance [27].
(3) BWAN: The Balanced and Weighted Alignment Network (BWAN) is a weighted alignment method for partial transfer tasks, aiming to alleviate negative transfer under target-domain missing-class conditions. It models the contributions of source samples and source categories in a weighted manner, suppressing the influence of source-private classes while enhancing the transfer of shared classes. BWAN considers both marginal and conditional distribution alignment and uses a balanced weighting strategy to improve the discriminability of shared classes in the target domain. Compared with global alignment methods, BWAN focuses more on filtering source-private classes and retaining shared classes, making it more suitable for partial domain adaptation scenarios [22].
(4) PADA: PADA is a partial domain adaptation method designed for incomplete label-space transfer. It estimates the relevance of source classes to the target domain and assigns different adaptation weights accordingly. By reducing the contribution of nontransferable classes, the method improves knowledge transfer and mitigates negative transfer effects [28].

All baseline models were evaluated under the same protocol used for MUAN. They shared identical input data, sample partitions, and preprocessing procedures, including signal processing and normalization. For each transfer task, the source domains, target domain, missing-class configuration, source training samples, and target adaptation samples were kept identical across methods. Missing classes were removed only from target-training data, while the test set always contained the full class space. Final performance was therefore measured on exactly the same independent test samples. Each baseline was independently trained five times using the same data-splitting protocol. The final results are summarized by mean accuracy and standard deviation across the five runs. The baseline methods were reimplemented following the architectures and optimization procedures described in their original publications, with only minor adjustments required to fit the unified multi-source experimental setting. Recommended hyperparameters from the original studies were used whenever applicable. When dataset-specific adjustment was required, only source validation data were used for parameter selection; labeled target test data were never involved. Table 3 presents the parameter settings of MUAN.

### 4.3. Experimental Results

#### 4.3.1. Simulation-Platform Dataset

In the experiments, the target-domain training set was assumed to miss specific classes, while all classes were available during testing. Table 4, Table 5, Table 6 and Table 7 report the fault localization results on the simulation-platform dataset under four transfer tasks and different missing-class settings. Overall, MUAN achieved the best performance across all tasks and all missing-class conditions, which demonstrates its stronger robustness and generalization capability under target-domain missing-class scenarios.

When the target domain contained all classes, MUAN effectively learned cross-domain shared features and maintained strong discriminative ability. When some middle-section classes are missing in the target-domain training set, conventional global alignment methods are more likely to suffer from label-space inconsistency, which aggravates negative transfer. This is mainly because DANN assumes identical label spaces in the source and target domains, and all source classes are aligned to the target domain during training. When some target classes are absent during training, source-private classes are still involved in the alignment process, which leads to incorrect transfer. For the rail insulation fault localization task considered in this study, different fault sections were highly continuous and similar, and the rail-potential waveforms of adjacent sections differed only slightly. As a result, incorrect alignment more easily blurred the class boundaries and degraded classification performance. Although MDFAN benefited from multi-feature fusion and showed some advantages in cross-condition feature extraction, its performance dropped rapidly in missing-class scenarios. This is because MDFAN mainly focuses on distribution shift but does not explicitly constrain label-space inconsistency. BWAN and PADA mitigate this problem to some extent through class weighting or partial alignment, but source-private classes can still contaminate the target feature space. In contrast, the proposed method further strengthens target-to-shared-space transfer through unilateral alignment, leading to better performance.

When six classes were missing from the target domain during training, the performance gap among methods was even larger. Compared with the best baseline BWAN, the proposed method improved accuracy by 15.73%, and it also outperformed PADA by 17.46%. This further confirms that the unilateral target alignment and source anchor constraints used in the proposed method are more effective for missing-class problems.

The standard deviations in Table 4, Table 5, Table 6 and Table 7 provide additional information about the run-to-run stability beyond the mean accuracy. MUAN showed relatively limited variation across the tested missing-class settings. Some baselines fluctuated more strongly on difficult transfer tasks, suggesting greater sensitivity to stochastic training and incomplete class coverage in the target-training data.

**Figure 6:**
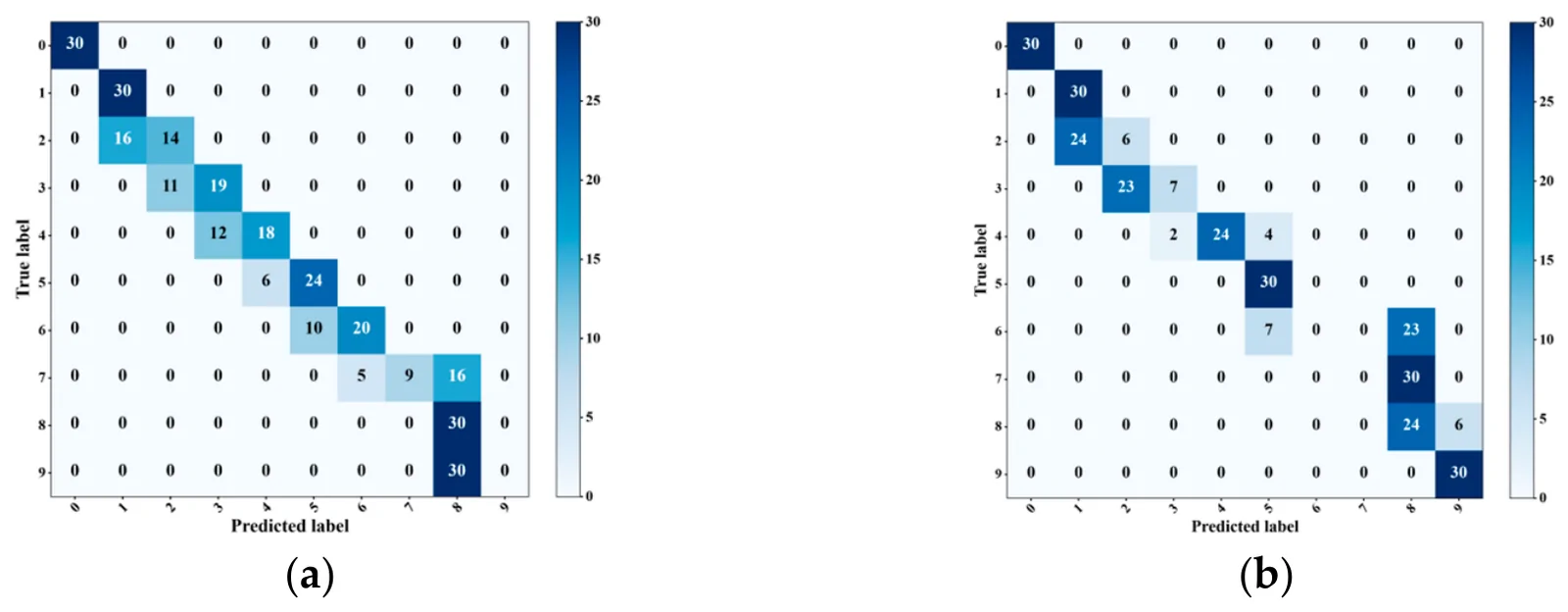

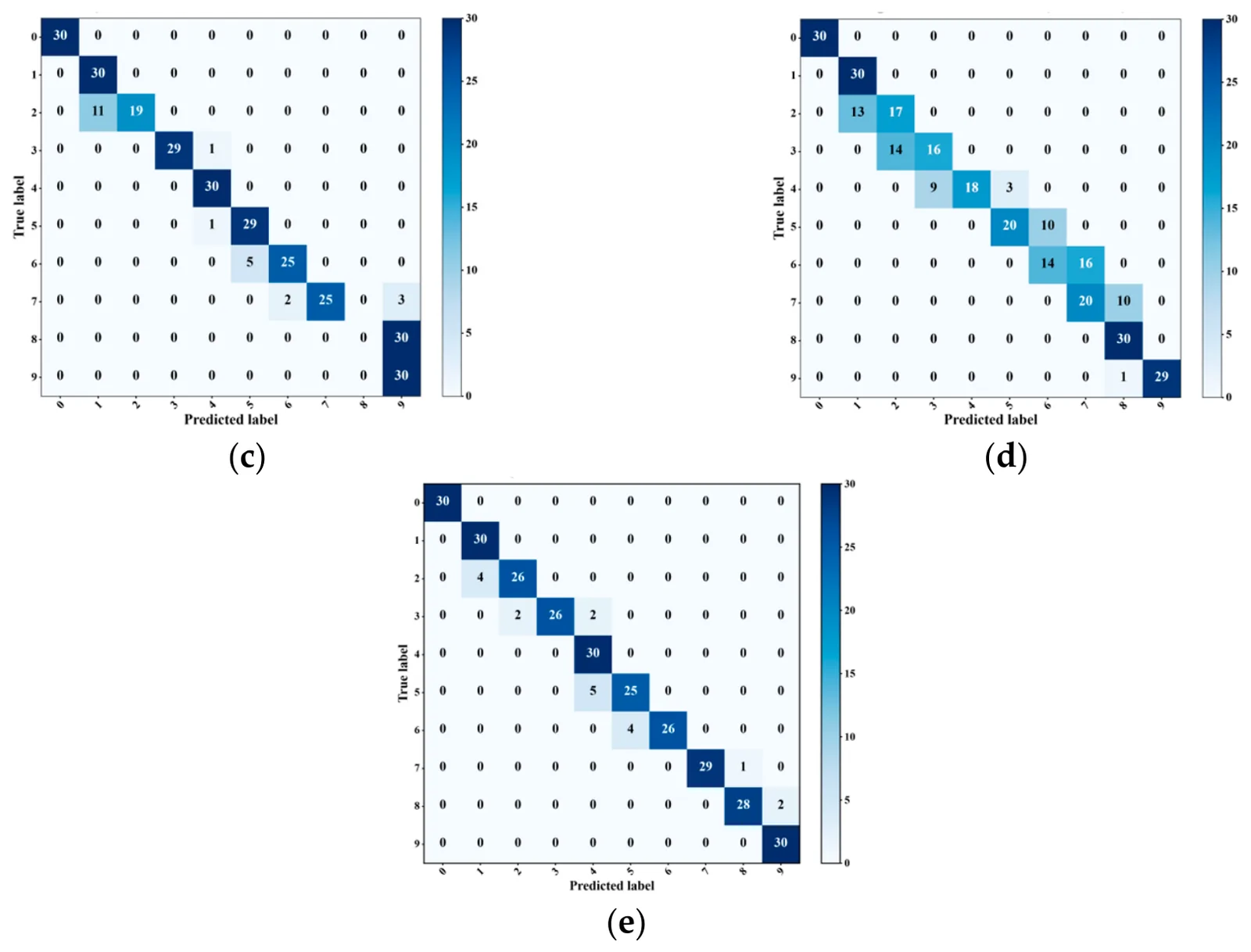
Confusion matrices on the simulation-platform dataset with the target domain set to 0.74P and classes 2–7 missing: (**a**) DANN, (**b**) MDFAN, (**c**) BWAN, (**d**) PADA, (**e**) MUAN.

The confusion matrices shown in Figure 6 show clear differences in fault localization performance when six fault classes were absent from the target-domain training set. For DANN and MDFAN, most misclassifications occurred between missing classes and adjacent fault locations, indicating that conventional domain alignment methods tend to force missing target samples toward incorrect source classes, which leads to obvious negative transfer. BWAN and PADA alleviate the mismatch to some extent, but confusion still remains at the missing fault locations, showing limited discrimination for missing-class boundaries and similar fault patterns. By contrast, MUAN achieved the highest fault localization accuracy of 93.33%. Classes 0, 1, 4, and 9 were classified perfectly, and the number of misclassified samples in the other classes was also greatly reduced. Only a few errors remained between adjacent fault classes. Compared with the baselines, MUAN effectively suppresses the negative transfer caused by missing classes, providing more stable and accurate fault localization.

**Figure 7:**
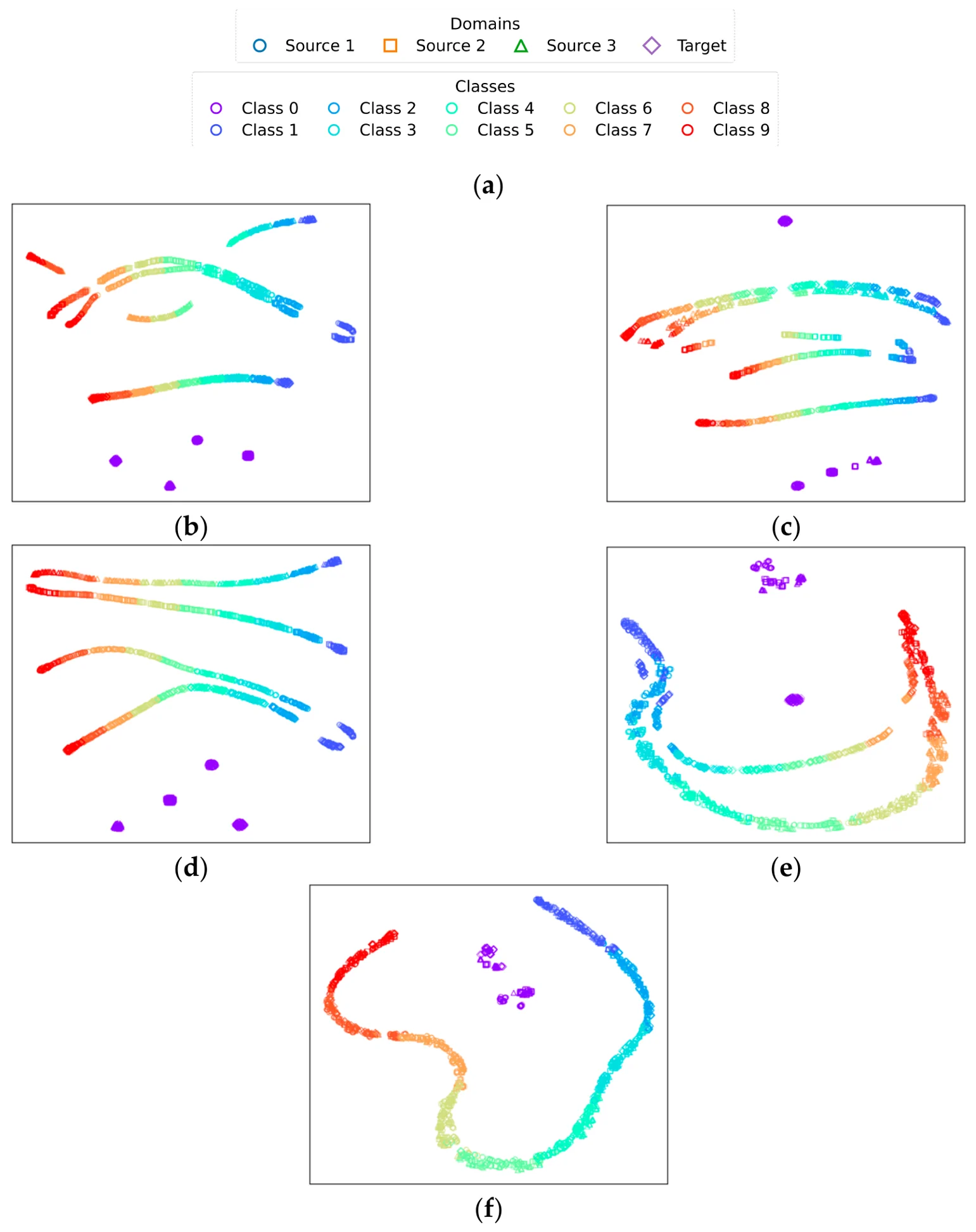
t-SNE feature distributions of the simulation-platform dataset with the target domain set to 0.74P and classes 2–7 missing: (**a**) legend, (**b**) DANN, (**c**) MDFAN, (**d**) BWAN, (**e**) PADA, (**f**) MUAN.

The t-SNE visualization shown in Figure 7 further supports these observations. In the case where six fault classes were missing from the target-domain training set, the feature spaces learned by DANN and MDFAN were highly scattered and partially mixed, with blurred boundaries between different classes. This indicates that global alignment alone is insufficient to handle negative transfer under incomplete label spaces. BWAN and PADA improved the feature structure to some extent, but some classes still overlapped, broke apart, or contained outliers, which shows that their ability to suppress missing-class interference is still limited. In contrast, the proposed approach generated a more discriminative feature representation, exhibiting tighter category-wise grouping and clearer separation among fault types. This suggests that the model can retain transferable diagnostic information while mitigating alignment errors caused by missing classes in the target-domain training set.

#### 4.3.2. Hardware-Platform Dataset

Table 8, Table 9, Table 10 and Table 11 summarize the results obtained from the hardware dataset. Compared with the simulation data, these measurements contain additional variations caused by circuit noise, measurement errors, and boundary fluctuations, producing more complex feature distributions. The hardware study therefore complements the numerical experiments by testing the method under more challenging laboratory conditions. MUAN retained high localization accuracy across the tested target-training missing-class settings.

Similar to the simulation results, DANN and MDFAN showed a clear performance drop under missing-class settings because they do not explicitly handle source-private classes. BWAN and PADA still achieved relatively good partial domain transfer performance on the hardware dataset. BWAN reduced the influence of irrelevant classes through a weighted alignment mechanism, improving shared-class alignment. PADA used class-weight screening to suppress source-private classes, alleviating the negative transfer caused by global alignment. However, both methods still aligned source and target features in a shared space. Their main objective was to reduce the overall distribution gap. When more classes were missing from the target-domain training set, this shared space was still affected by source-private classes, which compressed the feature distribution and weakened class separability.

In contrast, the proposed multi-source unilateral alignment strategy preserved the discriminative source structure and reduced private-class interference through source anchoring and directed target transfer. As a result, it showed better stability and robustness in complex cross-domain fault localization tasks.

**Figure 8:**
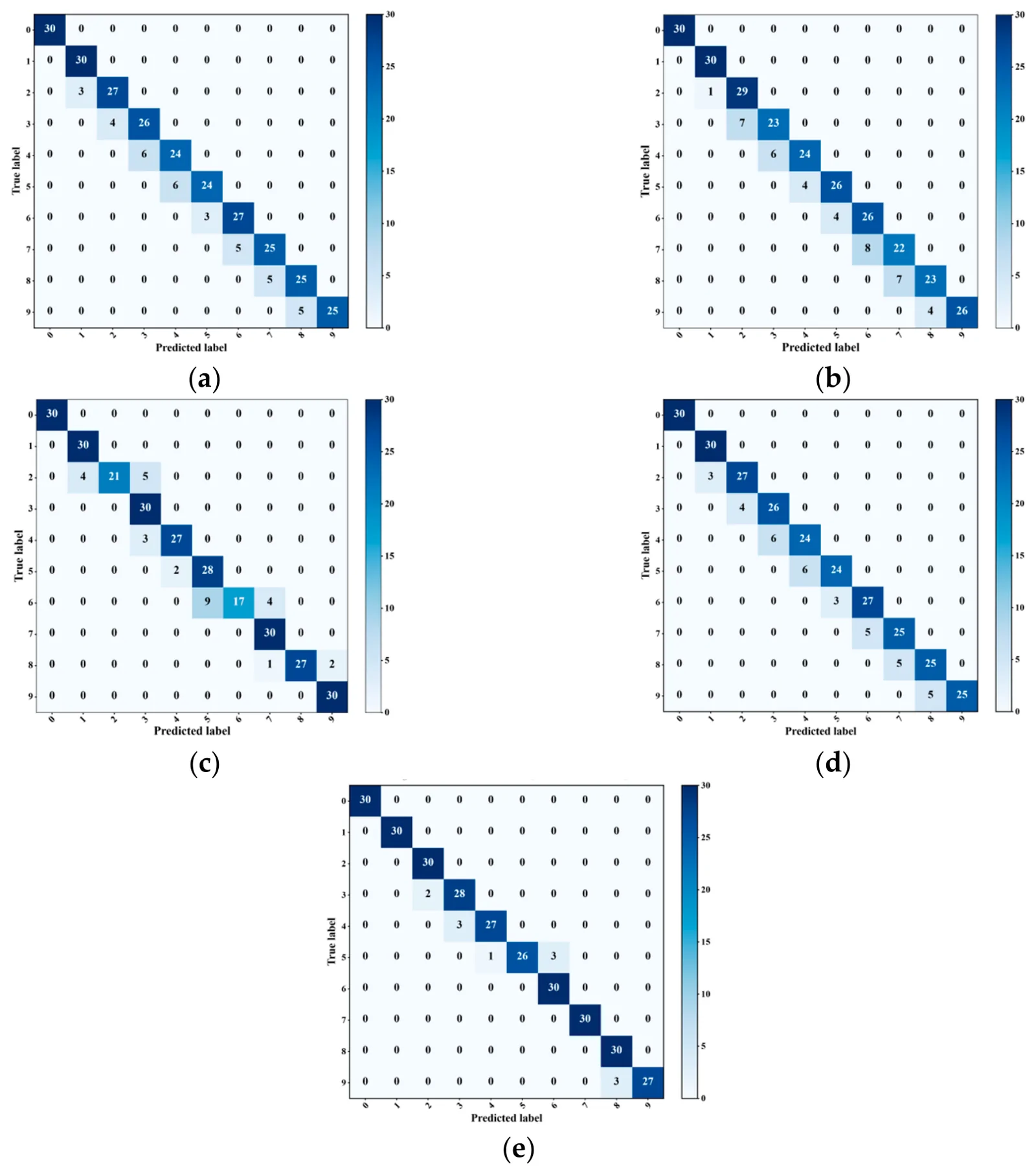
Confusion matrices of the hardware experimental platform dataset with the target domain set to 1.1P and classes 3–6 missing: (**a**) DANN, (**b**) MDFAN, (**c**) BWAN, (**d**) PADA, (**e**) MUAN.

The confusion matrix for the hardware dataset under the 1.1P condition with missing classes 3, 4, 5, and 6 is shown in Figure 8. It can be seen that MUAN achieved perfect recognition for classes 0, 1, 2, 6, 7, and 8. Only a few misclassifications occurred in classes 3, 4, 5, and 9, and most of them happened between adjacent fault locations. These results show that the proposed method can effectively suppress negative transfer when classes are missing from the target-domain training set, leading to more stable and accurate rail insulation fault localization.

**Figure 9:**
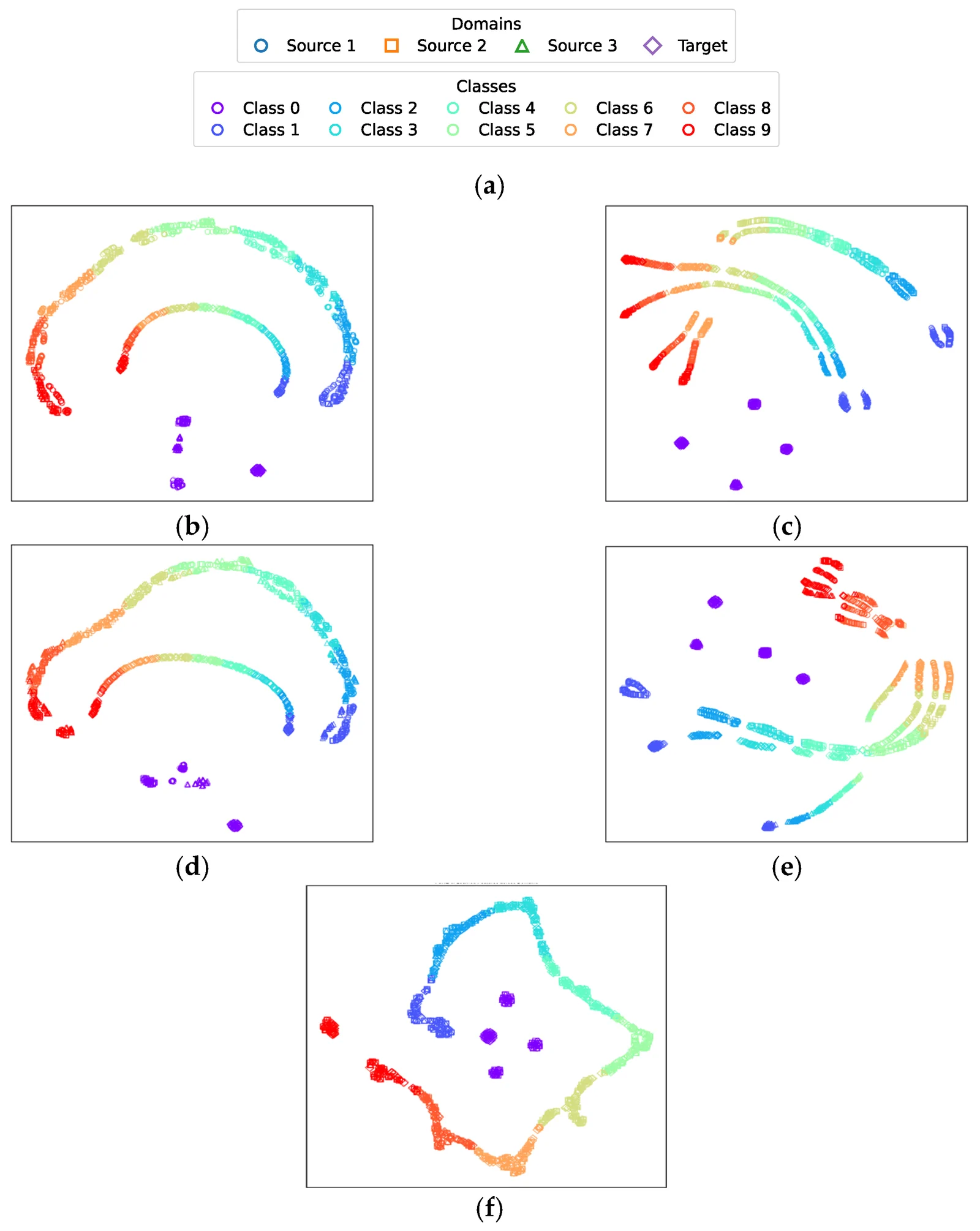
t-SNE visualization results of the hardware experimental platform dataset with the target domain set to 1.1P and classes 3–6 missing: (**a**) legend, (**b**) DANN, (**c**) MDFAN, (**d**) BWAN, (**e**) PADA, (**f**) MUAN.

The feature visualization in Figure 9 also shows that MUAN has strong feature extraction ability for grounding-fault localization. Samples from different classes overlap only slightly, while samples within the same class are well clustered.

Adaptation difficulty is determined not only by how many classes are missing but also by where those classes lie in the class space and how similar they are to the remaining classes. Different combinations may therefore lead to different levels of difficulty, and identical performance across all possible patterns is not assumed here. Nevertheless, MUAN remained competitive under the three substantially different settings considered in this study, including continuous, discrete, and severe continuous missing-class settings. Its performance also remained strong when six consecutive fault classes were unavailable. This suggests that the unilateral alignment mechanism is not restricted to one specific missing-class pattern. A comprehensive sensitivity study over all possible combinations will be considered in future work. The hardware platform should also be regarded as a laboratory-scale equivalent system rather than field validation using naturally occurring faults on an operating metro line.

To further evaluate the feasibility of online deployment, the model size and single-sample inference time of MUAN were compared with those of the baseline methods on an Intel(R) Xeon(R) CPU E5-2690 v3 @ 2.60 GHz platform. MUAN contained approximately 0.57 M parameters and required an average inference time of 1.50 ms/sample. In comparison, DANN contained approximately 0.04 M parameters, with an inference time of 0.91 ms/sample; PADA and BWAN each contained approximately 0.43 M parameters and required approximately 1.03 ms/sample; and MDFAN contained approximately 0.58 M parameters, with an inference time of 1.53 ms/sample. Although MUAN introduced a certain additional computational cost compared with some lightweight baseline methods, its inference latency remained at the millisecond level. Meanwhile, MUAN achieved higher and more stable fault localization accuracy under different operating conditions and target-domain training missing-class settings. Therefore, the proposed method provides a favorable trade-off between diagnostic performance and computational efficiency, and its inference efficiency is sufficient for practical online rail-insulation monitoring and fault localization.

#### 4.3.3. Ablation Study and Parameter Sensitivity Analysis

Four representative transfer tasks from the simulation and hardware datasets were selected to examine the contribution of the main MUAN components. The tasks spanned different target operating conditions and missing-class patterns. Four variants were tested by removing Stage 1 multi-source pretraining, L_a1_, the target correction and cross-domain alignment term L_a2_, and the adversarial loss L_d_. Table 12 reports the results. Every ablation caused a reduction in localization accuracy. The largest degradation occurred when Stage 1 pretraining or target correction with cross-domain alignment was removed, confirming the importance of stable multi-source knowledge and directional target alignment. Removing L_a1_ also weakened performance, showing the benefit of preserving the source feature structure during adaptation. A smaller but consistent decline appeared without adversarial learning, suggesting that it helped remove residual domain discrepancy after explicit alignment. The four components therefore contribute complementary functions to MUAN.

**Figure 10:**
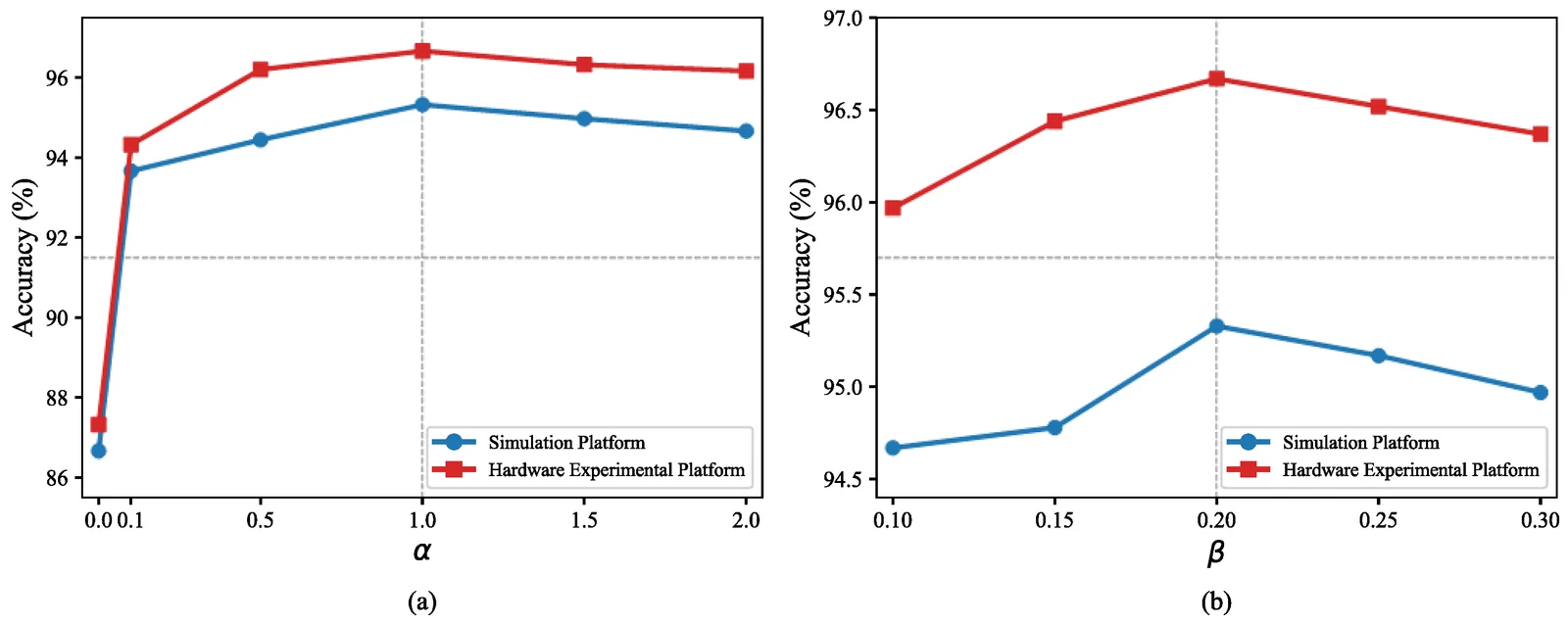
Parameter sensitivity analysis of MUAN on the simulation and hardware experimental datasets: (**a**) α; (**b**) β.

Parameter sensitivity was examined for α and β using the 1.1P target-domain task with classes 3–6 missing on both datasets. A one-factor-at-a-time procedure was used. For α, β was fixed at 0.2 and α ∈ {0, 0.1, 0.5, 1.0, 1.5, 2.0}. For β, α was fixed at 1.0 and β ∈ {0.10, 0.15, 0.20, 0.25, 0.30}. Figure 10 presents the results.

Setting α = 0 led to a clear accuracy reduction, confirming the importance of unilateral alignment. Accuracy rose as α increased and reached its maximum at 1.0 before decreasing slightly. The effect of β was less pronounced over the tested range, although both datasets achieved their best results at β = 0.20. Accordingly, α = 1.0 and β = 0.20 were adopted throughout the experiments.

## 5. Conclusions

This study develops MUAN, a two-stage transfer framework for rail insulation fault localization when the target-domain training data have incomplete class coverage. Through adversarial representation learning and directional adaptation, MUAN maintains the discriminative organization learned from the source domains while transferring target features toward a stable multi-source feature space. Stage 1 builds source anchors from labeled data. Stage 2 then uses SDA to preserve the source representation and applies directional target correction without forcing the source distribution to move toward an incomplete target space. This design limits interference from source-private classes while retaining source feature compactness and separability. Consequently, reliable localization can still be achieved even when multiple fault classes are unavailable during target adaptation.

The results from both the dynamic simulation and hardware platforms confirm the effectiveness of MUAN for cross-condition rail insulation fault localization. Across all evaluated tasks, the highest accuracies reach 98.33% and 99.67% on the simulation and hardware datasets, respectively. When only the three missing-class scenarios are considered, the corresponding average accuracies are 93.94% and 95.01%. Relative to the strongest baseline for each missing-class task, MUAN provides average gains of 6.84 and 4.67 percentage points on the two platforms. The largest improvement of 17.87 percentage points occurs for the severe missing-class case involving classes 2–7 on the simulation platform. These results indicate that unilateral alignment remains effective across operating-condition changes and incomplete target-training label spaces.

The hardware results should be interpreted within the scope of the present experimental setup. The platform is a laboratory-scale equivalent system rather than an operating metro line. It therefore provides additional evidence beyond numerical simulation but does not constitute direct field validation or demonstrate immediate deployability in a real metro network. Future studies will use field measurements from actual urban rail return systems, particularly data involving naturally occurring insulation degradation or grounding faults. Diagnosis of previously unseen fault modes will also be investigated to improve the practical reliability and diagnostic capability of MUAN.

## Figures and Tables

**Table 1:** Parameters of the MUAN modules.

Network Module	Layer	Kernel	Stride	Channel	Padding	Activation Function
Feature extractor	Conv1	3 × 1	(1, 1)	10	Yes	Sigmoid
	Average pooling	2 × 1	(2, 1)	10	Yes	/
	Flatten	5120	/	1	-	/
Feature classifier	FC1	256	/	1	/	Sigmoid
	FC4	256	/	1	/	ReLU
	FC5	10 or 5	/	1	/	Softmax
	FC6	1024	/	1	/	ReLU
Domain discriminator	FC7	1024	/	1	/	ReLU
	FC8	2	/	1	/	Softmax
CDA module	FC2	100	/	1	/	ReLU
	FC3	256	/	1	/	/

**Table 2:** Data labels.

Fault Type	Label
normal state	0
fault Section 1: 6201 m~6220 m	1
fault Section 2: 6221 m~6240 m	2
……	……
fault Section 9: 6361 m~6380 m	9

**Table 3:** Detailed parameter settings of MUAN.

Parameter	Value	Parameter	Value
Batch size	64	Epochs	1000
Stage 1 lr	1 × 10^−3^	Stage 2 lr	1 × 10^−4^
α β	1 0.2	*K*	3

**Table 4:** Fault localization accuracy (%) of different methods under different missing-class settings on the simulation-platform dataset with the target domain set to 0.8P (mean ± standard deviation).

	Missing Class	None	3, 4, 5, 6	1, 3, 6, 8	2–7
Model					
DANN		96.53 ± 1.30	80.20 ± 1.66	84.47 ± 3.26	63.00 ± 3.46
MDFAN		97.67 ± 2.13	67.80 ± 3.42	81.73 ± 3.50	53.27 ± 3.99
BWAN		97.33 ± 1.22	92.33 ± 1.76	94.53 ± 2.78	77.20 ± 3.08
PADA		96.93 ± 0.92	89.53 ± 2.74	92.33 ± 2.38	75.47 ± 3.31
**proposed method**		**98.33 ± 0.62**	**93.33 ± 0.53**	**95.27 ± 0.78**	**92.93 ± 0.76**

**Table 5:** Fault localization accuracy (%) of different methods under different missing-class settings on the simulation-platform dataset with the target domain set to 0.74P (mean ± standard deviation).

	Missing Class	None	3, 4, 5, 6	1, 3, 6, 8	2–7
Model					
DANN		95.13 ± 1.73	84.33 ± 2.95	86.60 ± 2.48	64.07 ± 3.16
MDFAN		97.07 ± 1.14	65.07 ± 1.06	84.40 ± 1.48	60.33 ± 3.37
BWAN		97.60 ± 1.12	91.60 ± 1.86	94.33 ± 2.11	82.67 ± 3.15
PADA		96.27 ± 1.67	85.67 ± 2.36	93.33 ± 2.39	74.80 ± 3.05
**proposed method**		**98.07 ± 0.49**	**94.60 ± 0.43**	**94.67 ± 0.85**	**94.07 ± 1.26**

**Table 6:** Fault localization accuracy (%) of different methods under different missing-class settings on the simulation-platform dataset with the target domain set to 1.1P (mean ± standard deviation).

	Missing Class	None	3, 4, 5, 6	1, 3, 6, 8	2–7
Model					
DANN		93.67 ± 1.15	81.67 ± 1.92	88.07 ± 2.75	71.60 ± 3.45
MDFAN		96.53 ± 0.93	58.13 ± 2.26	89.27 ± 2.33	60.07 ± 4.21
BWAN		96.67 ± 0.75	84.33 ± 2.07	93.87 ± 1.22	75.33 ± 3.35
PADA		96.27 ± 1.89	83.67 ± 2.15	89.07 ± 2.56	74.67 ± 3.09
**proposed method**		**96.67 ± 0.82**	**95.33 ± 1.20**	**95.93 ± 1.16**	**93.20 ± 1.28**

**Table 7:** Fault localization accuracy (%) of different methods under different missing-class settings on the simulation-platform dataset with the target domain set to 1.2P (mean ± standard deviation).

	Missing Class	None	3, 4, 5, 6	1, 3, 6, 8	2–7
Model					
DANN		91.27 ± 1.34	80.13 ± 2.82	84.33 ± 1.99	77.67 ± 3.07
MDFAN		96.93 ± 1.94	76.53 ± 2.24	89.40 ± 3.02	65.40 ± 4.16
BWAN		95.67 ± 0.89	90.07 ± 1.83	87.93 ± 1.52	79.53 ± 2.41
PADA		95.20 ± 1.51	84.27 ± 1.62	85.27 ± 2.27	76.27 ± 2.84
**proposed method**		**98.13 ± 0.61**	**91.60 ± 0.55**	**96.27 ± 1.32**	**90.07 ± 0.83**

**Table 8:** Fault localization accuracy (%) of different methods under different missing-class settings on the hardware experimental dataset with the target domain set to 0.8P (mean ± standard deviation).

	Missing Class	None	3, 4, 5, 6	1, 3, 6, 8	2–7
Model					
DANN		93.67 ± 1.58	80.33 ± 1.87	88.60 ± 3.43	77.33 ± 4.12
MDFAN		97.93 ± 1.36	87.07 ± 2.23	90.33 ± 3.17	82.33 ± 3.44
BWAN		95.93 ± 0.95	91.47 ± 2.71	91.93 ± 2.50	87.60 ± 3.47
PADA		94.33 ± 1.65	90.40 ± 2.58	91.20 ± 2.19	83.40 ± 4.43
**proposed method**		**96.33 ± 0.71**	**94.67 ± 0.88**	**95.33 ± 0.58**	**93.93 ± 1.09**

**Table 9:** Fault localization accuracy (%) of different methods under different missing-class settings on the hardware experimental dataset with the target domain set to 0.74P (mean ± standard deviation).

	Missing Class	None	3, 4, 5, 6	1, 3, 6, 8	2–7
Model					
DANN		93.00 ± 2.16	80.13 ± 1.98	80.33 ± 2.47	70.33 ± 4.55
MDFAN		98.33 ± 1.08	85.33 ± 3.10	88.33 ± 2.62	82.33 ± 3.62
BWAN		96.33 ± 1.11	91.80 ± 1.97	90.60 ± 1.82	87.33 ± 2.29
PADA		93.20 ± 2.05	85.60 ± 3.18	89.87 ± 2.18	81.60 ± 3.19
**proposed method**		**98.93 ± 0.60**	**95.67 ± 0.67**	**96.33 ± 0.53**	**93.27 ± 0.89**

**Table 10:** Fault localization accuracy (%) of different methods under different missing-class settings on the hardware experimental dataset with the target domain set to 1.1P (mean ± standard deviation).

	Missing Class	None	3, 4, 5, 6	1, 3, 6, 8	2–7
Model					
DANN		91.53 ± 1.74	87.67 ± 2.07	84.73 ± 2.46	74.60 ± 3.11
MDFAN		97.93 ± 1.69	82.73 ± 3.25	90.73 ± 2.91	83.53 ± 4.49
BWAN		94.33 ± 0.52	90.27 ± 1.37	93.67 ± 2.17	89.67 ± 2.81
PADA		95.93 ± 1.06	88.00 ± 2.45	90.87 ± 1.37	86.80 ± 3.13
**proposed method**		**99.67 ± 0.41**	**96.67 ± 0.87**	**96.00 ± 0.94**	**93.67 ± 1.18**

**Table 11:** Fault localization accuracy (%) of different methods under different missing-class settings on the hardware experimental dataset with the target domain set to 1.2P (mean ± standard deviation).

	Missing Class	None	3, 4, 5, 6	1, 3, 6, 8	2–7
Model					
DANN		92.87 ± 1.82	86.27 ± 2.68	88.93 ± 3.06	66.73 ± 3.69
MDFAN		97.67 ± 1.33	82.73 ± 3.03	86.27 ± 1.39	82.33 ± 3.81
BWAN		94.07 ± 1.38	91.87 ± 2.69	91.07 ± 1.62	86.73 ± 3.23
PADA		92.27 ± 1.19	89.07 ± 3.27	90.33 ± 2.24	83.40 ± 3.49
**proposed method**		**99.27 ± 0.54**	**94.60 ± 0.78**	**96.27 ± 0.72**	**93.67 ± 1.73**

**Table 12:** Ablation results of MUAN under representative transfer tasks (%).

Model	Simulation 0.8P Classes 2–7	Simulation 1.1P Classes 1, 3, 6, 8	Hardware 0.74P Classes 3, 4, 5, 6	Hardware 1.2P Classes 2–7	Average
w/o Stage 1	86.20	89.73	90.27	88.40	88.65
w/o La1	90.07	93.07	92.33	90.87	91.59
w/o La2	87.13	88.47	89.13	86.80	87.88
w/o Ld	91.20	92.60	93.33	91.80	92.23
MUAN	92.93	95.93	95.67	93.67	94.55

## Data Availability

The data that support the findings of this study are available from the corresponding author upon reasonable request.
